# Protection of Sensitive Loads in Distribution Systems Using a BSFCL-DVR System

**DOI:** 10.3390/s21051615

**Published:** 2021-02-25

**Authors:** Mehdi Firouzi, Saleh Mobayen, Hossein Shahbabaei Kartijkolaie, Mojtaba Nasiri, Chih-Chiang Chen

**Affiliations:** 1 Department of Electrical Engineering, Faculty of Engineering, Abhar Branch, Islamic Azad University, Abhar 1584743311, Iran; 2 Advanced Control Systems Laboratory, Department of Electrical Engineering, University of Zanjan, University Blvd., Zanjan 4537138791, Iran; 3 Future Technology Research Center, National Yunlin University of Science and Technology, 123 University Road, Section 3, Douliou, Yunlin 64002, Taiwan; 4 Golestan Provinace Distribution Electrical Company, Gorgan 517212, Iran; Hossein_1798@yahoo.com; 5 Solar Energy Applications Group, Trinity College Dublin, University of Dublin, D02 PN40 Dublin, Ireland; nasirim@tcd.ie; 6 Department of Systems and Naval Mechatronic Engineering, National Cheng Kung University, Tainan 70101, Taiwan; ccchenevan@mail.ncku.edu.tw

**Keywords:** dynamic voltage restorer, fault current limiter, sensitive loads, power quality, voltage controller

## Abstract

In this paper, an incorporated bridge-type superconducting fault current limiter (BSFCL) and Dynamic Voltage Restorer (DVR) is presented to improve the voltage quality and limiting fault current problems in distribution systems. In order to achieve these capabilities, the BSFCL and DVR are integrated through a common DC link as a BSFCL-DVR system. The FCL and DVR ports of the BSFCL-DVR system are located in the beginning and end of the sensitive loads’ feeder integrated to the point of common coupling (PCC) in the distribution system. At first, the principle operation of the BSFCL-DVR is discussed. Then, a control system for the BSFCL-DVR system is designed to enhance the voltage quality and limit the fault current. Eventually, the efficiency of the BSFCL-DVR system is verified through the PSCAD/EMTDC simulation.

## 1. Introduction

The expansion and complexity of modern power systems, as well as emergent technologies and power quality problems, make the design and engineering of distribution systems challenging [1]. Among all power quality problems, momentary outages, and voltage sag are the most important ones. For sensitive loads, even a shallow voltage dip can cause malfunction and operation failure [2,3]. Furthermore, the most voltage sags mainly occur due to the distribution system downstream short circuit faults [4]. The distribution system topology is one of the main factors for voltage sag depth and duration. A large fault current in a feeder of parallel feeders results in voltage sag at the point of common coupling (PCC), which affects the loads in other parallel feeders connected to the PCC. The main adverse effects of voltage sags are [5,6,7,8,9]:

- Malfunction of protection equipment
- Tripping of process control and desktop computers
- Costly production wastage due to interruption of process materials
- Malfunction of wind turbine grid-side converters

Therefore, researchers are studying techniques to suppress the fault current and reduce the voltage sag under fault condition [8,9,10]. It is generally less costly to tackle the problem at its lowest level, i.e., close to the load. In [11], a dual-function dynamic voltage restorer (DVR) is proposed to mitigate voltage sag and fault current level in distribution system. To achieve this capability, the flux-charge feedback-based control system is implemented to the DVR, which acts as large inductance to limit downstream fault currents. In [12], a dual-functional bridge-type fault current limiter (BTFCL) is presented and designed, which can operate in both current limiting modes to reduce faulty currents and DVR mode to compensate the voltage sag in distribution system. In [13], a new FCL-DVR scheme is proposed, which can operate with different protection strategies under grid disturbances. It includes a crowbar bidirectional-thyristor switch, which is located in the output of DVR to limit the fault currents. All design considerations of the FCL-DVR parameters including power rating of DVR converter, output filter reactors and the DC link capacitor are presented. In [14], a novel FCL-DVR concept is proposed, which both capabilities of limiting fault current and compensating the PCC voltage. The main advantage of proposed FCL-DVR concept is its reduced number of components compared to the other FCL-DVR schemes. In [15], an improved FCL-DVR scheme with energy-optimized control strategy is proposed, which has both capabilities of other FCL-DVR schemes with the capacity optimization of its key components. 

In [16], a voltage-booster scheme includes the DVR and high-temperature superconducting FCL (HTS-FCL) are designed and implemented to the doubly-fed induction generator (DFIG) for enhancing the fault-ride through (FRT) performance. In [17], the unified inter-phase power controller (UIPC) is used to control power flow in hybrid AC/DC micro-grid. In [18], a multi-step braking resistor is proposed to enhance the FRT performance of fixed-speed wind turbines. In [19], a multi-resistor BFCL (MRBFCL) is proposed to mitigate the PCC voltage under different voltage sag levels. In this configuration multi resistors has been used instead of single resistor in the conventional BFCL in [20]. It is implemented to the DFIG [19] and permanent-magnet synchronous generator (PMSG)-based [19] wind turbines for enhancing the FRT, respectively. Authors in [21] presents a dynamic multi-cell BFCL to compensate the PCC voltage of wind farms. It is connected between the wind farm and grid without using coupling transformer and can compensate the PCC voltage under whole voltage sag level. In [22], a superconducting-based FCL and a MW class DVR integrated with superconducting-magnetic energy-storage (SMES) system are presented for voltage sag compensation in distribution system. Considering this background, this paper proposes a new solution based on superconductive and power electronic devices for limiting fault current and compensating the PCC voltage in distribution systems.

Several solutions based on superconductive and power electronic devices have been reported in [23,24,25,26,27,28,29,30]. In [29], a combination of series phase shifter (SPS) with SMES has been suggested to enhance the transient stability in the power system. In [24], a combination of SMES with Static synchronous compensator has been used for improving wind turbine generation system stability. In [25], the combined SMES and solid-state phase shifter have been proposed to enhancement the control of the system. In [26], a novel combined device has been presented, which is connected in series with two independent power systems. The voltage regulation is the main function of this device. In [27,28], a combined superconducting fault current limiter (SFCL) and SMES has been proposed to suppress the fault current and improve the transient behavior in a microgrid.

**Figure 1:**
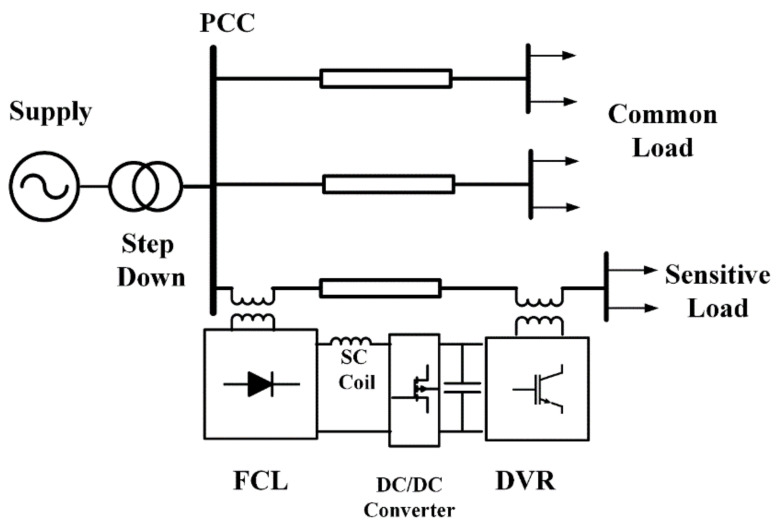
BSFCL-DVR system.

In this paper, a bridge-type SFCL (BSFCL) and a dynamic voltage restorer (DVR) are combined to create new dual-functional equipment as a BSFCL-DVR system for limiting the fault current and improvement of the voltage quality. The schematic diagram of the BSFCL-DVR is shown in Figure 1. It is connected at the beginning and end of the feeder supplying a sensitive load. The following desired features can be mentioned for the proposed structure:

- The superconducting coil can be used as an energy storage device in the DVR and limiting element in BSFCL. The proposed BSFCL-DVR system needs only one superconducting coil.
- The BSFCL only limits the transient fault current at the beginning time of fault and cannot limit the amplitude of the fault current. However, by using the BSFCL-DVR system, the fault current amplitude is limited as well as transient fault current.
- The DVR of the proposed structure can compensate steady-state load voltage under both normal and fault conditions.
- It can compensate sensitive load voltages during fault and prevent from a system outage.
- The voltage quality at the PCC is compensated; therefore the loads on parallel feeders would not affect during the fault period.
- The proposed BSFCL-DVR system increases the reliability of sensitive loads.

However, the disadvantages of the BSFCL-DVR system are high compensation capacity voltage source inverter (VSI) for the DVR port and additional communication with the protection system as fault detection function. In this paper, the power circuit and principle operation of the BSFCL are presented in Section 2. The brief description of DVR is presented in Section 3. In Section 4, the proposed structure is given. Section 5 proves the new BSFCL-DVR performance by comparing it in three cases on the PSCAD/EMTDC. Eventually, the conclusions of this study are presented in Section 5. 

## 2. Bridge-Type SFCL

Different types of FCL including SFCLs, solid state FCLs (SSFCLs) and resonant-type FCLs (RFCLs) have been proposed and documented in literature [29,30]. The application of SFCLs was found as promising solution to limit fault current and also can increase the reliability of system [31], enhance the power quality in distribution system [32], mitigate the transformer inrush currents [33], mitigate the transient over voltages [34], and improve the power system transient stability [35]. They are classified in to three resistive-type, inductive-type and bridge-type SFCL (BSFCL). This bridge-type SFCL consists of high-temperature superconducting coil (HTSC) and a diode bridge rectifier. Superconductors are used to limit fault current in both AC and DC grids with significant advantages such as compact size and high efficiency [36,37]. The BSFCL has specific characteristics including:

- It can suppress the transient fault current at the beginning time of fault without any delay;
- It requires any fault detection and controller system;
- It prevents from happening instantaneously deep voltage drop at the beginning time of the fault.

The major disadvantage of BSFCL is that it cannot stop gradually increase of the fault current.

### 2.1. Power Circuit of Bridge-Type SFCL

In Figure 1, the BSFCL power circuit is represented. It includes the following parts:

- Three coupling transformers (*T_a_*, *T_b_*, and *T_c_*).
- Three-phase bridge rectifier (D_1_–D_6_) to converts the AC line current to DC current (*i_d_*)
- A superconducting coil (SC) is connected to the DC side of the bridge rectifier circuit as limiting impedance [38,39,40,41].

### 2.2. BSFCL Characteristic 

**Figure 2:**
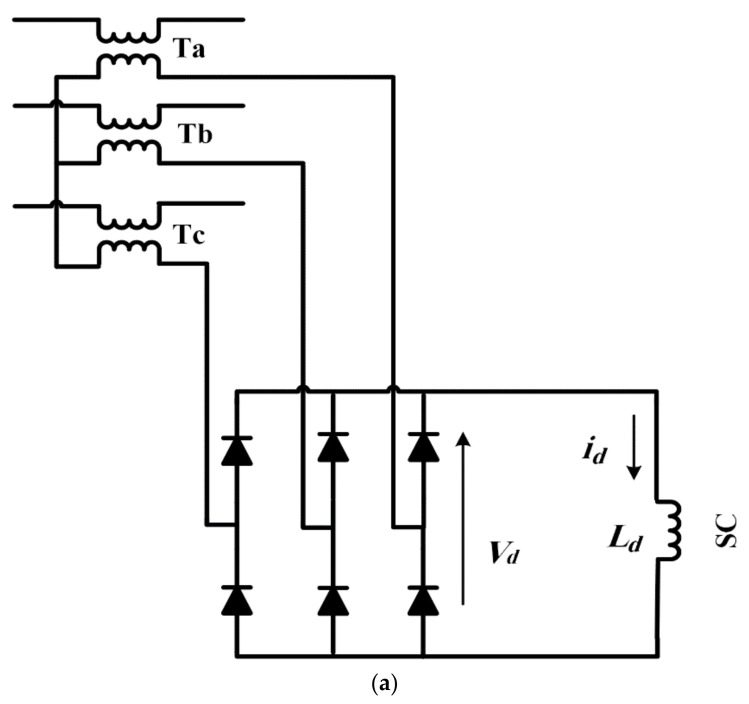

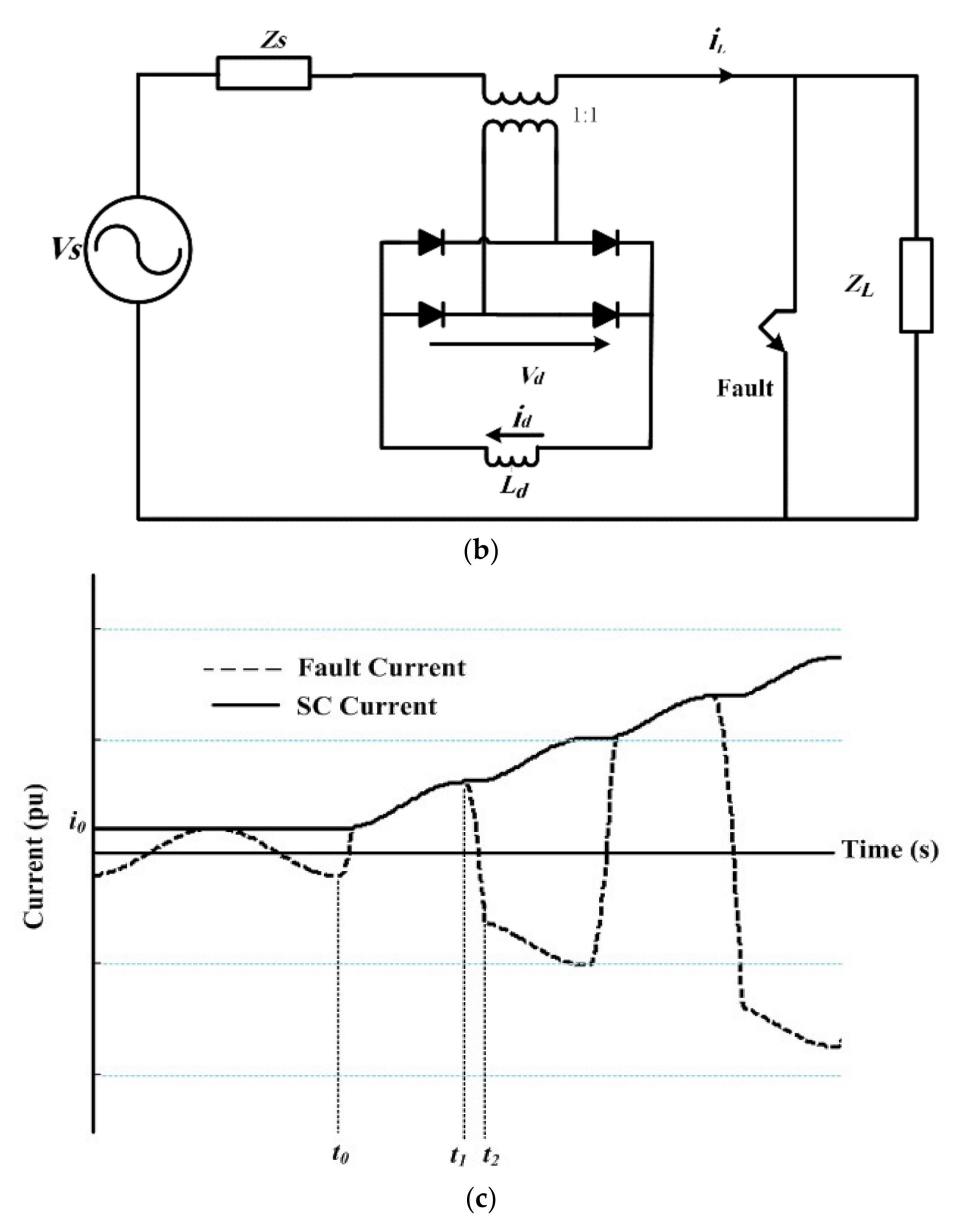
(**a**) BSFCL circuit; (**b**) test system for analytical analysis; (**c**) SC and fault current.

Figure 2b shows the test circuit, which is employed for analytical studies. The source and load impedances are presented by$\;Z_{s}=r_{s}+j\omega L_{s}$ and $Z_{L}=r_{L}+j\omega L_{L}$. The transformer turn ratio is assumed to be 1. Figure 2c demonstrates the line and SC currents in the presence of the BSFCL. As demonstrated in this figure, the current of the SC is approximately constant in the normal condition and, we have:

(1) $$V_{d}=L_{d}\frac{di_{d}}{dt}=0$$

Therefore, the BSFCL does not affect the line side under this condition. Once, a short circuit fault happens at *t = t*_0_, the SC current (*i_d_*) and subsequently the line fault current (*i_L_*) starts to increase with a constant rate. The performance of the BSFCL is divided into two periods. In the first period *t*_0_ *< t < t*_1_, both SC and line currents are equal. The KVL law to obtain the line current (*i_L_*) is given by:

(2) $$\sqrt{2\;}V\;sin\left(\omega t\right)=ri_{L}+\;L\frac{di_{L}}{dt}$$

where$\;r=r_{s}$ and $L=L_{s}+L_{d}$. Considering (2), the *i_L_*, is determined, as follows:

(3) $$i_{L}=e^{\frac{r}{L}\left(t-t_{0}\right)}\left\{i_{0}-\frac{\sqrt{2}V}{Z}\sin\left(\omega t-\varphi \right)\right\}+\frac{\sqrt{2}V}{Z}\sin\left(\omega t-\varphi \right)$$

where, $i_{0}=i_{L}\left(t=t_{0}\right)$, $Z=\sqrt{r^{2}+{\left(L\omega \right)}^{2}}$ and $tan\left(\varphi \right)=L\omega /r$. In the second period *t*_1_ *< t < t*_2_, the *i_d_* is higher than the *i_L_*. Therefore, the *i_d_* flows through the diodes. The currents *i_d_* and *i_L_* are presented as follows:

(4) $$i_{d}=e^{\frac{r}{L}\left(t-t_{1}\right)}\left(i_{1}\right)$$

and

(5) $$i_{L}=e^{\frac{r}{L}\left(t-t_{1}\right)}\left\{i_{1}-\frac{\sqrt{2}V}{Z}\sin\left(\omega t-\varphi \right)\right\}+\frac{\sqrt{2}V}{Z}\sin\left(\omega t-\varphi \right)$$

where $i_{1}=i_{L}\left(t=t_{1}\right)$, $Z=\sqrt{r^{2}+{\left(L\omega \right)}^{2}}$, $tan\left(\varphi \right)=L\omega /r$,$\;r=r_{s}$, and $L=L_{s}$.

## 3. Dynamic Voltage Restorer

**Figure 3:**
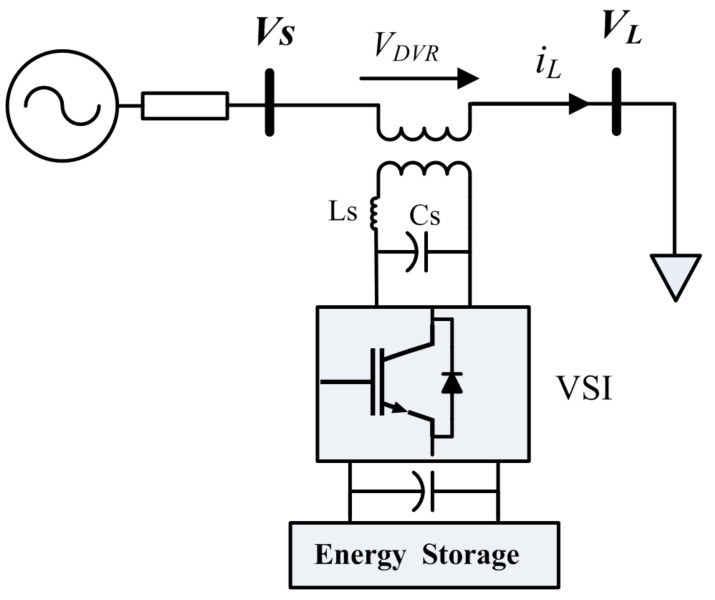
Power circuit of DVR.

Figure 3 represents the DVR power circuit. It can protect the sensitive loads from destructive effects of voltage drop. It essentially consists of an injection transformer, a low pass filter to eliminate the high order harmonics, which represented by *L_f_* and *C_f_*, a voltage source inverter (VSI), and an energy storage system (ESS). In this paper, the pulse-wide-modulation (PWM) technique is used to control the VSI. To restitute the load voltage at the pre-fault time, the DVR will inject a voltage to compensate the voltage drop. In order to increase the voltage, the active power capacity of the ESS is a limiting factor. This is important, especially for mitigating long duration voltage sag [42,43]. To compensate the voltage sag by the DVR, several control techniques have been proposed in the literature. They are classified into pre-sag [42], in-phase [43], and minimal energy [44] methods. 

The sensitive loads characteristic is the main factor to determine the control method. In the in-phase control method, the VDVR is injected in-phase with source-side voltage (Vs), regardless of the pre-fault voltage and the load current. The amplitude of the injection voltage is the main advantage of this method [42,43]. In the minimal energy method, the injected voltage by the DVR is perpendicular to the load current. Therefore, it does not require active power to raise the voltage [44,45]. The nonlinear loads are vulnerable to phase jumps in the voltage drop condition [44]. The pre-sag compensation strategy is applied to overcome this problem by increasing the instantaneous load voltage to the pre-sag voltage phase and magnitude [42]. 

## 4. Proposed Structure

**Figure 4:**
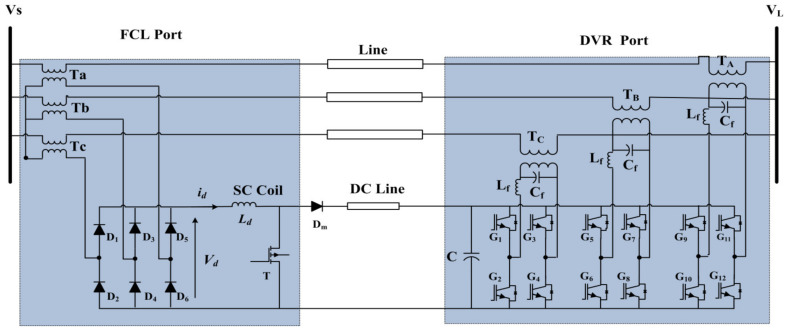
Proposed structure.

Figure 4 shows the BSFCL-DVR power circuit. It is mainly composed of three single-phase VSIs (i.e., IGBT *G*_1_–*G*_4,_ *G*_5_–*G*_8_, and *G*_9_–*G*_12_), inverter output filter (*L_f_* − *C_f_*), bridge rectifier circuit (*D*_1_–*D*_6_), a boost regulator (MOSFET *T* and diode *D_m_*), SC coil (*L_d_*). DC link (*C*), which is located between the DVR and boost converter. The DVR is located into the load side through three boost transformers (*T_A_*, *T_B_*, *T_C_*) at the end of the feeder, which are the DVR function port. Transformers (*T_a_*, *T_b_*, *T_c_*) are integrated into the SC coil via a three-phase bridge rectifier circuit as an FCL port. Under the operation mode of the grid, the T is closed and the DVR port is used to compensate the load voltage. The current following through the SC is approximately constant, so the FCL port does not effect on the line side. 

**Figure 5:**
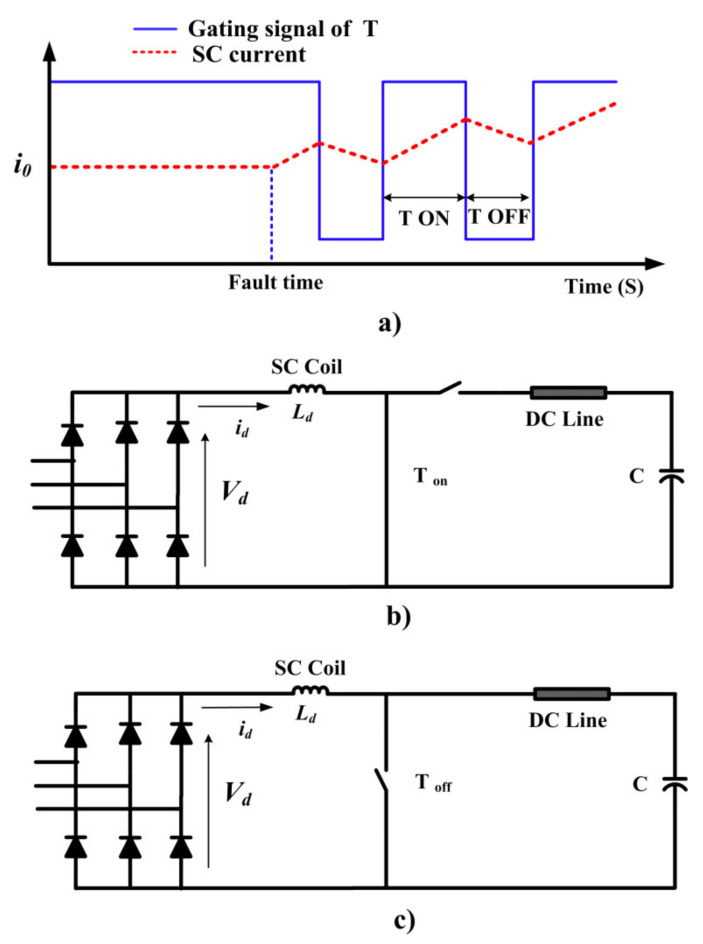
Operation of chopper in the fault condition, (**a**) gating signal of T, (**b**) T is ON, and (**c**) T is OFF.

Figure 5 shows the gating signal of the *T*, operation of the boost chopper for charging the capacitor when *T* is on and off states under fault condition, respectively. In the fault condition, the circuit operation is divided into two modes. The first mode is shown in Figure 5b. In this mode, T is on and the input voltage across the SC, i.e., *V_d_* is equal to the voltage across the SC and *i_d_* linearly increases, which leads to raising of energy in the SC. In the second mode, the T is turned off and the SC current flows through the diode (*D_m_*) as demonstrated in Figure 5c. Also, some of the SC energy is transferred to the capacitor and *i_d_* linearly decreases as shown in Figure 5a. By appropriate switching of *T*, the DC link voltage remains constant. Simultaneously, the DVR converter injects the AC voltage to the line at the end of the feeder at the load side.

### Control Strategy

**Figure 6:**
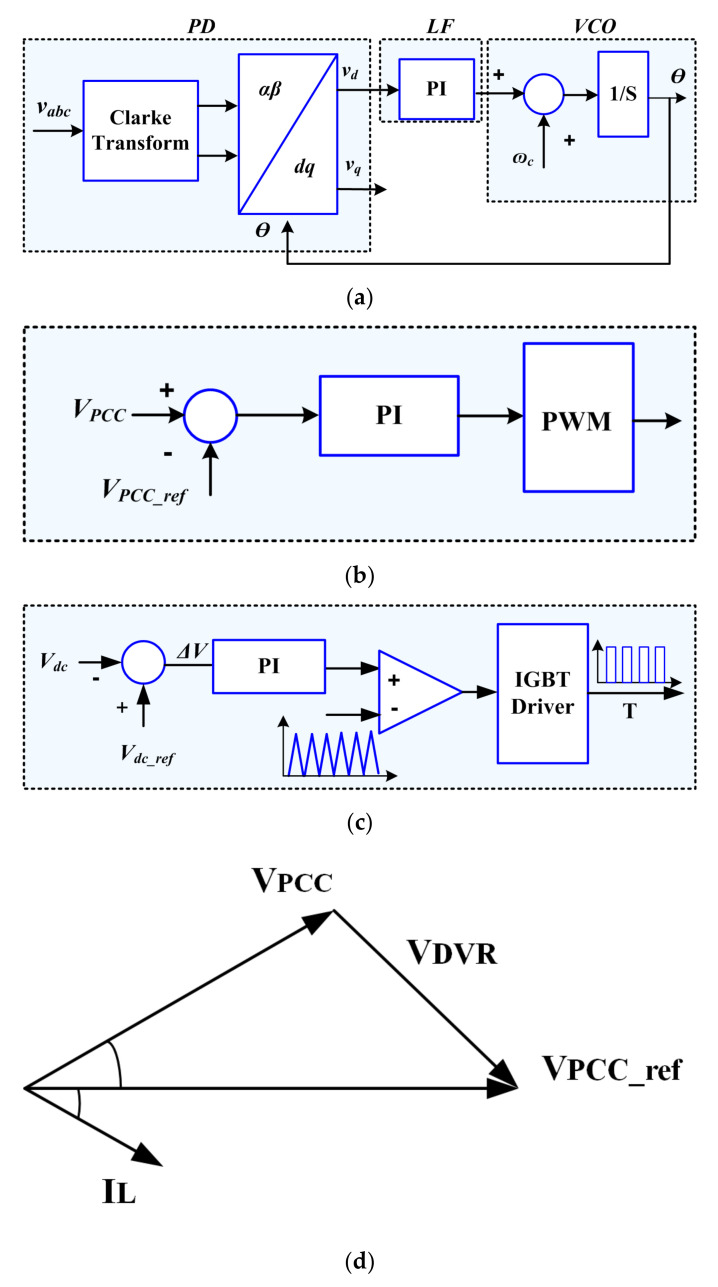
(**a**) PLL diagram, (**b**) Control strategy DVR during fault, (**c**) Control strategy of the boost chopper during fault, (**d**) phasor diagram of DVR voltage injection.

The main functions of the new structure are limiting fault current and compensate the load voltage during a fault condition. The DVR port restored the load voltage to its desired level. In this paper, the phase-locked loop (PLL) synchronization method has been used. Figure 6a demonstrates the block diagram of the PLL, which includes the voltage-controller oscillator (VCO), a phase detector (PD), and a loop filter (LF). Under the fault condition, the PCC voltage (*V_PCC_*) is controlled based on the pre-sag compensation strategy. Figure 6b demonstrates the control scheme of the DVR port. The main drawback of this method is the capacity limitation of the ESS to support active power for compensation the voltage sag. But, the BSFCL-DVR system solves this drawback. In the proposed method, the boost converter provides constant DC voltage for the capacitor with absorbing the SC energy stored. Figure 6c,d show the control scheme of the boost chopper regulator and the phasor diagram of the DVR port. 

## 5. Simulation Results

**Figure 7:**
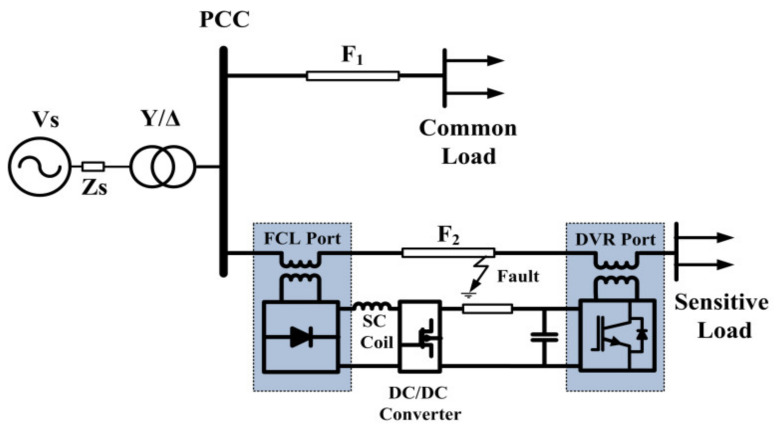
Simulated system.

The simulated system is shown in Figure 7. Simulation studies were performed in PSCAD/EMTDC software to investigate the efficiency of the BSFCL-DVR system. The parameters of the studied system are given in Table 1. 

Simulations have been performed for three following cases:

Case A: No FCL is in operation,
Case B: Conventional BSFCL is in operation and,
Case C: DVR-BSFCL is employed.

### 5.1. Case A

**Figure 8:**
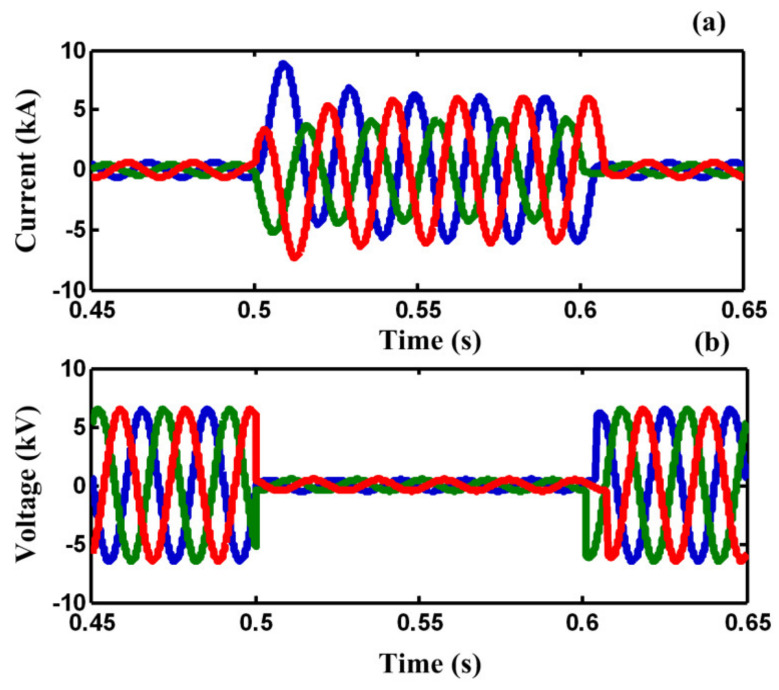
(**a**) Fault current in case A, (**b**) voltage of PCC in case A.

In case A, a three-line-to-ground (3LG) fault happens at *t =* 0.5 *s* on feeder 2 (F_2_). Figure 8a,b demonstrate the line current of F_2_ and the PCC voltage without using any FCL. As illustrated in Figure 8a,b, the fault current is increased to the peak value of 8 kA and the PCC voltage falls to zero, approximately. 

### 5.2. Case B

**Figure 9:**
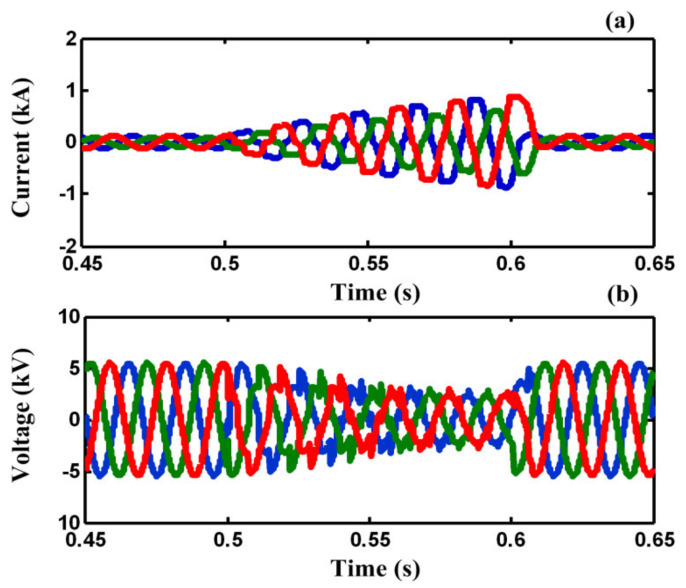
(**a**) Fault current in case B, (**b**) voltage of PCC in case B.

Figure 9a,b demonstrate the line current of F_2_ and the PCC voltage with using conventional BSFCL in the mentioned fault condition. As demonstrated in Figure 9a, that the transient fault current is limited, however, the fault current increases gradually during the fault period. Also, it causes the PCC voltage sag gradually to increase. To compare the harmonic of *V_PCC_*, Table 2 is presented. As shown in Table 2, in case B (conventional BSFCL), The THD of *V_PCC_* is 25%. Additionally, it also includes both even and odd harmonics. Furthermore, the amplitude of the fundamental component is low. It is quite obvious that the conventional BSFCL can limit the fault current; however, it cannot satisfy the requirements of the IEEE 519 standard for the PCC voltage. However, the sensitive loads on F_2_ experience power outage during the fault.

### 5.3. Case C

**Figure 10:**
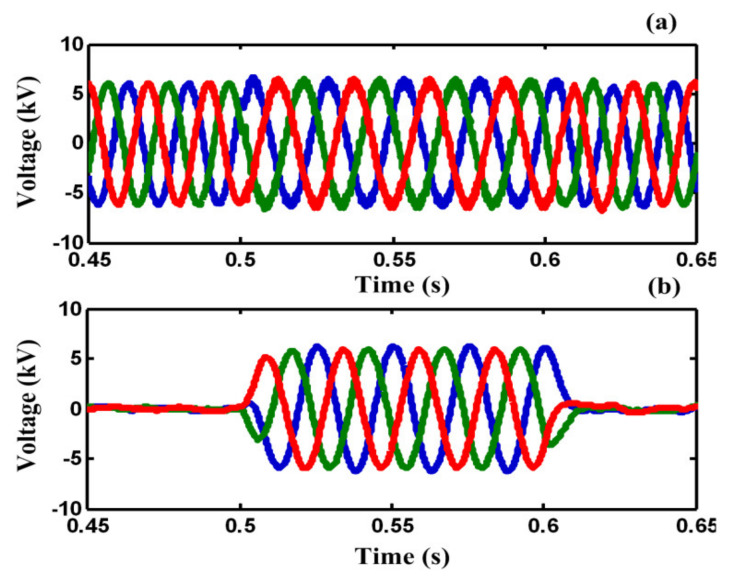

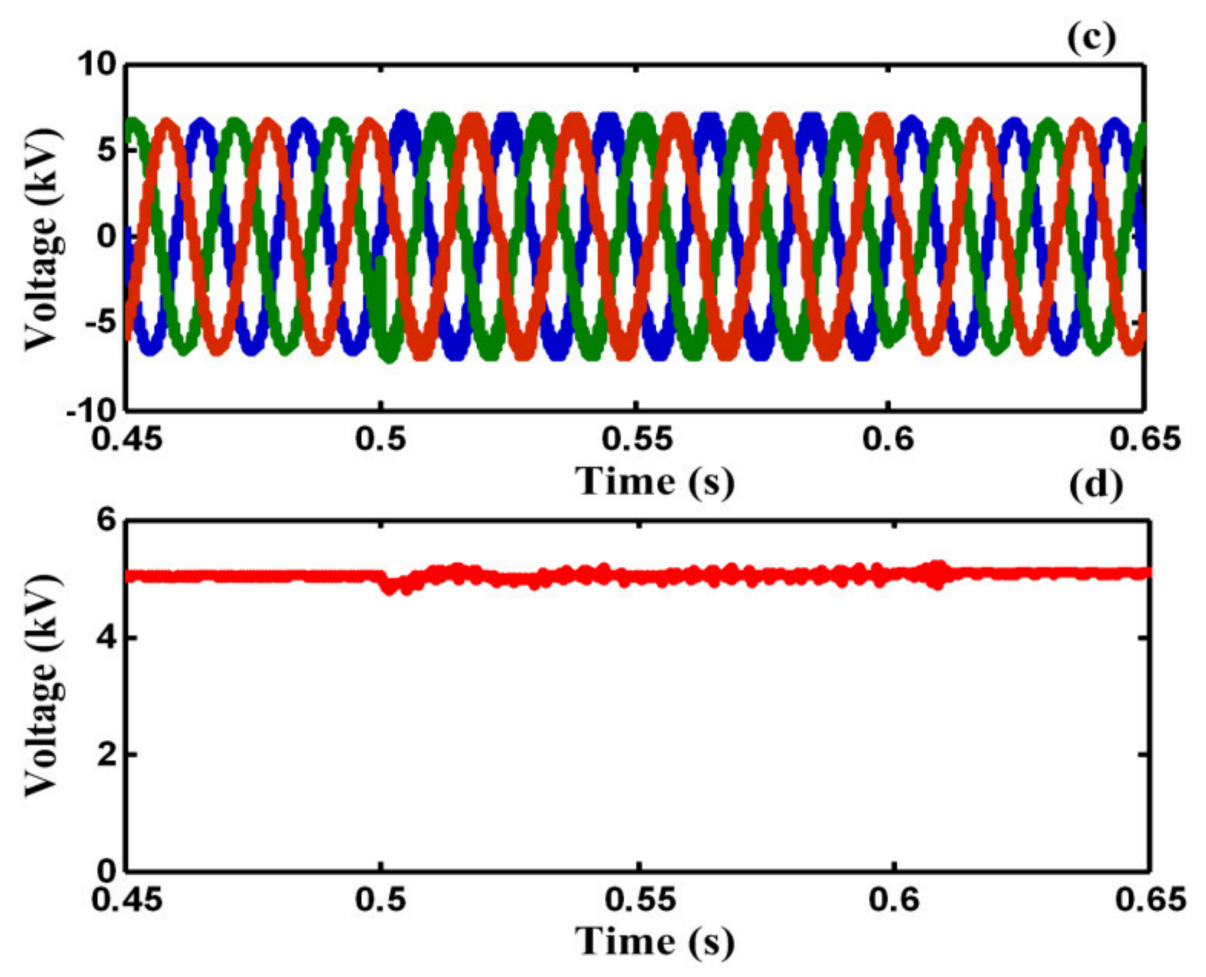
(**a**) load voltage, (**b**) DVR port injected voltage, (**c**) PCC voltage and, (**d**) capacitor voltage during fault, in case C.

Figure 10a,b illustrate the load voltage and the injected voltage by DVR port in F_2_ during the fault, respectively. As shown in Figure 10a, by using the proposed DVR-BSFCL structure, the load voltage is compensated to pre-fault voltage in F_2_. Figure 10c,d show PCC voltage and capacitor voltage, respectively. Although the PCC voltage is distorted during the fault, it is protected from voltage sag. 

**Figure 11:**
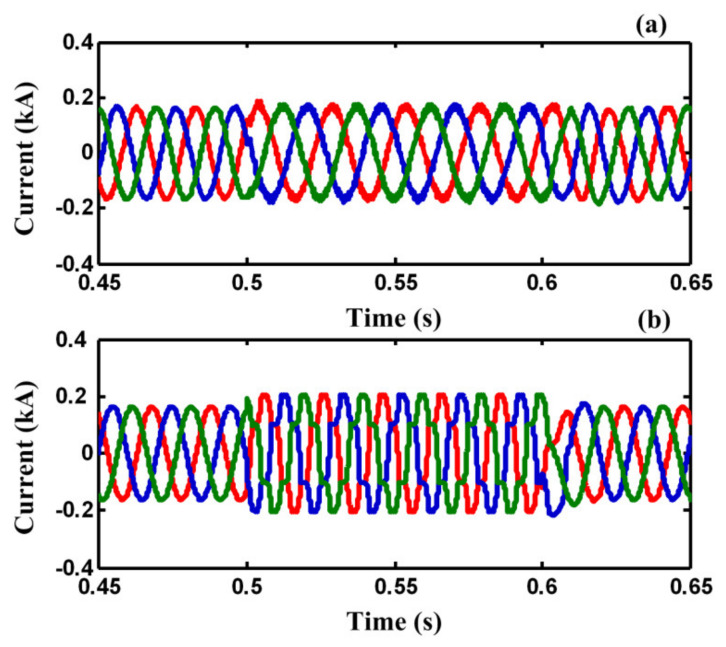
(**a**) The load current and, (**b**) fault current of faulted feeder (F2) during fault operation mode in case C.

**Figure 12:**
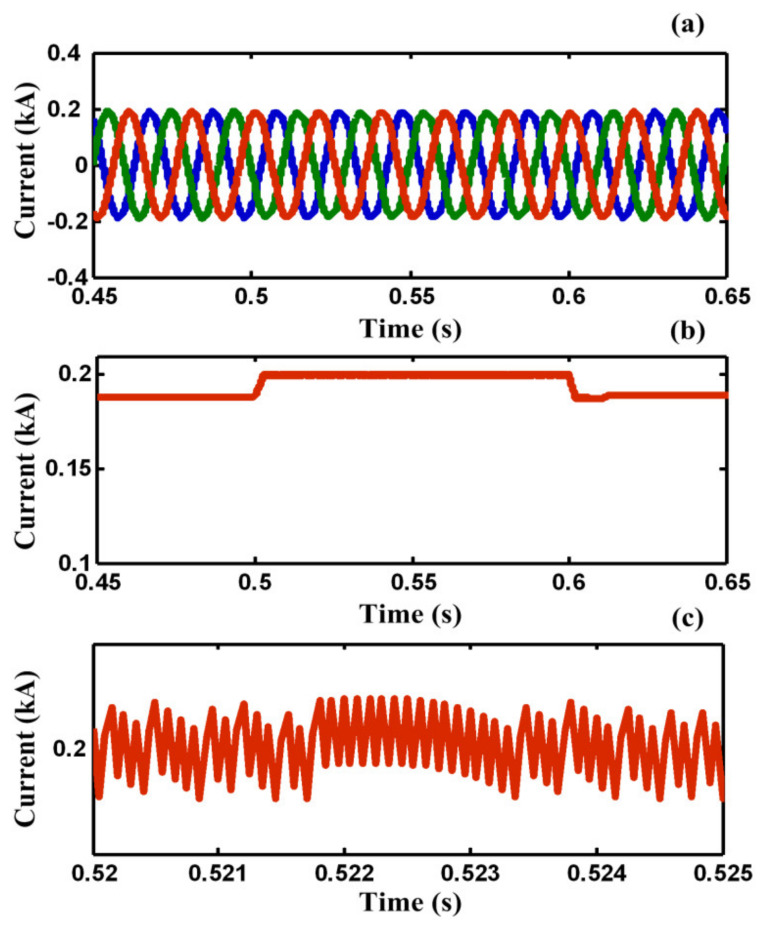
(**a**) The current of common loads in F_1_, (**b**) SC coil current, (**c**) enlargement SC coil current during fault in case C.

Figure 11 shows the load and fault currents of F_2_. As shown in Figure 11a, the load current would not be affected by fault occurring and it is protected from momentary outages during the fault. However, the fault current in the F_2_ effectively is limited by the FCL port and is not increased gradually during the fault. As shown in Table 2, the relative amplitude of harmonic components of PCC voltage in case C is much less than case B. Figure 12a shows the current of common loads connected to PCC during the fault, respectively. It is obvious from Figure 12a that the load current of F_1_ is protected from voltage sag and momentary outage during the fault. Figure 12b,c show the SC coil current and enlargement SC current during fault, respectively.

## 6. Conclusions

This paper presents a new combined BSFCL-DVR system to protect the PCC voltage and limits fault current in distribution including multi feeder connected to the PCC bus. It requires only one superconducting energy storage, which reduces the cost of using this device in distribution system. It has two FCL and VDR ports, which is interlined between the beginning and end of feeders. In the normal condition, the FCL port does not affect system performance and DVR port compensates the load voltage. In the fault condition, the fault current is effectively limited by FCL port and the voltage of sensitive loads have been compensated in pre-fault voltage by DVR port, simultaneously. Additionally, the PCC voltage remains at an acceptable level and the other connected loads to the PCC continue their safe operation. 

## Figures and Tables

**Table 1:** Parameters of study system.

Parameters		Value
**Grid**	Supply voltage(Vs)	20 kV
	Frequency (f)	50 Hz
	Rs	0.05 Ω
	Ls	1 mH
	Step down transformer	20 kV/6.6 kV 5 MVA
**Line**	Line resistance (R)	0.2 (Ω/km)
	Line reactance (X)	0.4 (Ω/km)
	Length of feeder1	20 km
	Length of feeder ^2^	10 km
**Load**	Impedance of common load	1 MVA, PF = 0.9 lag
	Impedance of sensitive load	1 MVA, PF = 0.8 lag
**Proposed Structure**	DVR port transformers	4 kV/4 kV, 2 MVA
	Capacitor	4 kV,1000 uF
	SC inductance	100 mH
	L_f_	25 uH
	C_f_	50 uF
	frequency switching of chopper	2 kH

**Table 2:** Comparison of harmonic components of PCC voltage in cases B and C.

Order of Harmonics	Case B	Case C
1	0.5839	1
2	0.0289	0.0001
3	0.0088	0.0001
4	0.0155	0.0001
5	0.1087	0.0363
6	0.0303	0.0001
7	0.0489	0.0301
8	0.0219	0.0001
9	0.0121	0.0006
10	0.0162	0.0001
11	0.0381	0.0261
12	0.0189	0.0001
13	0.0316	0.0214
14	0.0282	0.0001
15	0.0431	0.0086
**THD**	**25.76%**	**6.9%**

## Data Availability

The data that support the findings of this study are available within the article.
